# Erhöhtes Risiko eines COVID-19-bedingten Krankenhausaufenthaltes für Arbeitslose: Eine Analyse von Krankenkassendaten von 1,28 Mio. Versicherten in Deutschland

**DOI:** 10.1007/s00103-021-03280-6

**Published:** 2021-01-28

**Authors:** Morten Wahrendorf, Christoph J. Rupprecht, Olga Dortmann, Maria Scheider, Nico Dragano

**Affiliations:** 1 Institut für Medizinische Soziologie, Centre for Health and Society, Medizinische Fakultät, Universität Düsseldorf, Moorenstraße 5, 40225 Düsseldorf, Deutschland; 2 Abteilung Gesundheitspolitik und Gesundheitsökonomie, AOK Rheinland/Hamburg – die Gesundheitskasse, Düsseldorf, Deutschland

**Keywords:** Gesundheitliche Ungleichheiten, COVID-19, Arbeitslosigkeit, Infektionskrankheiten, Sozialepidemiologie, Deutschland, Health inequalities, COVID-19, Unemployment, Infectious disease, Social epidemiology, Germany

## Abstract

**Hintergrund und Ziel:**

Arbeitslosigkeit steht in Zusammenhang mit Armut und ist ein Risikofaktor für schlechte Gesundheit. Der vorliegende Beitrag untersucht, ob Arbeitslosigkeit das Risiko für einen COVID-19-bedingten Krankenhausaufenthalt für Männer und Frauen im erwerbsfähigen Alter in Deutschland erhöht.

**Methoden:**

Die Auswertungen verwenden Krankenkassendaten der AOK Rheinland/Hamburg (vom 01.01.2020 bis zum 18.06.2020) mit Daten zu 1.288.745 Personen zwischen 18 und 65 Jahren. 4 Erwerbssituationen werden unterschieden: (1) reguläre Erwerbstätigkeit, (2) Niedriglohntätigkeit mit Sozialleistungen, (3) Arbeitslosigkeit mit Bezug von Arbeitslosengeld 1 (Alg I) und (4) Langzeitarbeitslosigkeit mit Bezug von Arbeitslosengeld 2 (Alg II). COVID-19-Krankenhausaufenthalte werden über Meldungen der Krankenhäuser anhand der ICD-Codes U07.1 und U07.2 bestimmt. Berechnet werden multiple logistische Regressionsmodelle (für Alter und Geschlecht adjustiert).

**Ergebnisse:**

1521 Personen hatten im Beobachtungszeitraum einen Krankenhausaufenthalt mit COVID-19 als Haupt- oder als Nebendiagnose. Dies entspricht insgesamt einer Rate von 118 Fällen pro 100.000 Versicherten. Die Raten variieren je nach Erwerbssituation. Im Vergleich zu regulär Erwerbstätigen liegt das Odds Ratio im Falle von Langzeitarbeitslosigkeit (Alg II) bei 1,94 (KI 95 %: 1,74–2,15), für Empfänger von Alg I bei 1,29 (KI 95 %: 0,86–1,94) und für Niedriglohnverdiener bei 1,33 (KI 95 %: 0,98–1,82).

**Schlussfolgerung:**

Die Ergebnisse stimmen mit früheren Studien aus den USA und Großbritannien zu sozioökonomischen Ungleichheiten bzgl. Risikos von COVID-19-Krankenhausaufenthalten überein. Dies liefert erste Hinweise dafür, dass sozioökonomische Unterschiede in Bezug auf schwere Verläufe von COVID-19 auch in Deutschland auftreten.

## Abstract (en)

**Background and Objective:**

Unemployment is related to poverty and is a risk factor for poor health. The present study investigates if unemployment increases the risk of COVID-19 hospitalization for men and women of working age in Germany.

**Methods:**

The study uses the health insurance data from AOK Rhineland/Hamburg (from 1 January 2020 until 18 June 2020) of 1,288,745 persons aged between 18 and 65. Four employment situations are distinguished: (1) regular employment, (2) low-wage employment with social support, (3) unemployment with receipt of unemployment benefit 1, and (4) long-term unemployment with receipt of unemployment benefit 2. COVID-19 hospitalizations are measured on the basis of the ICD codes U07.1 and U07.2 reported by the hospitals. Multiple logistic regression models are calculated (adjusted for age and sex).

**Results:**

During the observation period, 1521 persons had hospitalization with COVID-19 as primary or secondary diagnosis. Overall, this corresponds to a rate of 118 cases per 100,000 insured persons. Rates varied by employment situation. Compared with regularly employed persons, the odds ratio for a hospitalization was 1.94 (CI 95%: 1.74–2.15) for long-term unemployment, 1.29 (0.86–1.94) for unemployed, and 1.33 (0.98–1.82) for low-wage employment.

**Conclusion:**

The results are in line with earlier studies from the USA and Great Britain reporting socioeconomic inequalities in COVID-19 hospitalization risk. This provides the first empirical support that socioeconomic inequalities in the severity of COVID-19 also exists in Germany.

## Einleitung

Sozioökonomische Unterschiede spiegeln sich in Deutschland auch in der Gesundheit ganzer Bevölkerungsgruppen und deren Lebenserwartung wider. Eine aktuelle Untersuchung berichtet etwa [1], dass Männer mit einem niedrigen Einkommen durchschnittlich eine um 8,6 Jahre kürzere Lebenserwartung als Männer mit hohem Einkommen haben; bei Frauen sind es 4,4 Jahre. Diese sozialen Unterschiede (meist bestimmt anhand von Unterschieden in Einkommen, Bildung und beruflicher Position) sind in Deutschland und anderen Ländern auch für eine Vielzahl einzelner Erkrankungen wie Diabetes, Atemwegserkrankungen, koronare Herzkrankheiten und Depressionen gut belegt [2–4]. Auch im Falle von Infektionskrankheiten, wie viralen Erkrankungen der Atemwege, gibt es klare Hinweise auf sozioökonomische Unterschiede. Dies gilt beispielsweise für die H1N1-Pandemie im Jahre 2009/2010 und bei der saisonalen Influenza. Ein Zusammenhang zwischen einer benachteiligten sozioökonomischen Position (kurz „SEP“) findet sich dabei sowohl für ein grundsätzliches Infektionsrisiko als auch für unterschiedliche Indikatoren zur Schwere der Infektionserkrankung (z. B. Krankenhausaufenthalt, intensivmedizinische Versorgung, Beatmung oder Mortalität; [5–9]). Damit stellt sich die Frage, ob sozioökonomisch benachteiligte Bevölkerungsgruppen auch während der aktuellen Pandemie häufiger und schwerer an COVID-19 (Corona Virus Disease 2019) erkranken.

In der Tat deutet aktuell eine wachsende Zahl internationaler Studien darauf hin, dass sich sozioökonomisch benachteiligte Bevölkerungsgruppen häufiger mit dem Virus (SARS-CoV-2) infizieren und dass sie zugleich im Falle einer COVID-19-Erkrankung schwerere Krankheitsverläufe haben (zur Übersicht siehe: [10–12]). Dies zeigen zum Beispiel Studien aus England und den USA, die den Zusammenhang regionaler sozioökonomischer Indikatoren (z. B. durchschnittliches Einkommensniveau einer Region) und aggregierter Daten zu COVID-19 in der jeweiligen Region untersuchen (sogenannte ökologische Studien). Ein Vergleich von New Yorker Stadtteilen zeigt beispielsweise [13], dass zu Beginn der Pandemie die Zahl der SARS-CoV-2-Infektionen in ärmeren Stadtvierteln im Allgemeinen höher war als in reichen Stadtteilen. Ähnliches gilt für COVID-19-bedingte Krankenhausaufenthalte und Mortalität [14]. Das gleiche Muster zeigen auch Untersuchungen aus England, in denen detaillierte Informationen zum Ausmaß sozioökonomischer Deprivation in einzelnen Gebieten (verfügbar für mehr als 32.000 Gebiete) mit der durchschnittlichen Anzahl an COVID-19-Todesfällen verknüpft wurden [15–17]. Aktuell sind hierzu auch 2 Studien aus Deutschland erschienen, in denen 401 Landkreise und kreisfreie Städte verglichen werden. Dabei zeigt sich, dass sozioökonomisch benachteiligte Gebiete zwar zu Beginn der Pandemie weniger Erkrankungen aufwiesen, doch mit zunehmender Dauer der Pandemie stärker betroffen waren [18]. Zusätzliche Auswertungen zeigen, dass diese Umkehrung vor allem für Gebiete im Süden Deutschlands gilt, die bereits früh hohe Infektionszahlen aufwiesen [19]. Eine wichtige Einschränkung dieser ökologischen Studien besteht allerdings darin, dass sie auf unterschiedlichen Aggregationsebenen basieren und dass weitreichende Schlussfolgerungen auf individueller Ebene nicht möglich sind (aufgrund der Gefahr ökologischer Fehlschlüsse).

Bislang liegt nur eine kleine Anzahl von Studien vor, die sich direkt auf individuelle Daten stützen können. Auswertungen auf Basis der UK-Biobank (Kohortenstudie) zeigen zum Beispiel, dass sich Personen ohne Bildungsabschluss doppelt so häufig mit SARS-CoV‑2 infizierten wie Personen mit Hochschulabschluss [20]. Unter Verwendung derselben Daten ergab eine weitere Studie ein erhöhtes Risiko eines COVID-19-bedingten Krankenhausaufenthaltes für Personen mit niedrigem Haushaltseinkommen [21]. Darüber hinaus zeigen amtliche Daten aus England und Wales deutliche Unterschiede der COVID-19-Mortalität nach beruflicher Position, mit den höchsten Risiken für unqualifizierte Berufe und Dienstleistungsberufe [22]. 2 weitere Studien stammen aus den USA und vergleichen COVID-19-Krankenhauspatienten mit der übrigen Bevölkerung. Sie zeigen einerseits, dass Patienten mit Krankenhausaufenthalt ein geringeres Einkommen als die Allgemeinbevölkerung haben [23] und dass COVID-19-Krankenhauspatienten häufiger EmpfängerInnen von staatlicher finanzieller Unterstützung (i.e. „Medicaid“ und „Medicare“) sind – Fürsorge, die in den USA nur Personen mit geringem Einkommen erhalten können [24].

Für Deutschland liegen vergleichbare populationsbasierte Studien mit Individualdaten zum jetzigen Zeitpunkt hingegen noch nicht vor. Das Ziel des vorliegenden Beitrags ist es daher, auf Basis von Daten der Gesetzlichen Krankenkasse von mehr als 1 Mio. versicherten Männern und Frauen die Forschungsevidenz für Deutschland zu verbessern. Damit einhergeht auch die Frage, ob die oben berichteten Ungleichheiten in den USA und Großbritannien auch für andere Länder (mit anderen Wohlfahrts- und Gesundheitssystemen) bestehen. Hierzu werden COVID-19-bedingte Krankenhausaufenthalte als Indikator für schwere Krankheitsverläufe analysiert. Die Bestimmung sozioökonomischer Benachteiligung erfolgt anhand verfügbarer Details zur Erwerbssituation, durch die Arbeitslosigkeit und der Bezug von Transferleistungen ermittelt werden können. Arbeitslosigkeit und der Bezug von Transferleistungen stellen einen wichtigen Ansatz zur Bestimmung von Armut in der Forschungsliteratur dar (sogenannter politisch-normativer Ansatz; vgl. [25]) und gelten als wichtige Indikatoren für eine sozioökonomische Benachteiligung. Arbeitslosigkeit steht zudem in mehrfacher Hinsicht mit der Gesundheit in Zusammenhang, z. B. über psychosozialen Stress, materielle Deprivation oder armutsbedingtes ungesundes Verhalten, und ist ein bekannter Risikofaktor für zahlreiche chronische Erkrankungen [26, 27]. Es kann somit vermutet werden, dass die Wahrscheinlichkeit einer schweren COVID-19-Erkrankung mit erforderlichem Krankenhausaufenthalt unter Arbeitslosen im Vergleich zu regulär Beschäftigten erhöht ist.

## Methoden

### Datenbasis

Die vorliegende Studie verwendet Krankenkassendaten der AOK Rheinland/Hamburg, eine der größten Krankenkassen in Deutschland mit Versicherten v. a. in den Regionen Rheinland und Hamburg. Insgesamt liegen für den Beobachtungszeitraum vom 01.01.2020 bis zum 18.06.2020 Daten zu 2.768.417 Versicherten vor, die für die Analyse wie folgt eingeschränkt werden: Erstens werden die Analysen auf Männer und Frauen zwischen 18 und 65 Jahren begrenzt, um ausschließlich Personen im erwerbsfähigen Alter zu vergleichen. Zweitens beschränken sich die Analysen auf Personen mit verfügbaren Informationen zur Erwerbssituation, die als aktiv am Arbeitsmarkt gelten. Hierzu gehören sowohl Personen, die gegenwärtig erwerbstätig sind, als auch solche, die arbeitslos, aber erwerbsfähig und auf Arbeitssuche sind. Entsprechend diesen Einschränkungen werden auch Studierende, RentenbezieherInnen (inkl. Erwerbsminderungsrente) und Personen, die weder erwerbstätig noch arbeitssuchend sind (z. B. nichterwerbstätige Partner von erwerbstätigen Versicherten), ausgeschlossen. Mit Stand 17.07.2020 ergibt sich aus diesen Einschränkungen eine Studienpopulation von 1.288.745 Personen (570.034 Frauen; 718.711 Männer).

### Messung

#### *COVID-19-bedingter Krankenhausaufenthalt*:

Im Falle eines Krankenhausaufenthaltes erfasst das zuständige ärztliche Personal Haupt- und Nebendiagnosen, die täglich an die Versicherung übermittelt werden. Ein Krankenhausaufenthalt aufgrund von COVID-19 wird durch die internationalen WHO ICD-10-GM-Codes U07.1 (laborbestätigt) und U07.2 (symptombezogen, negativer oder kein Labortest) angezeigt. In dem seltenen Fall, dass mehr als ein Krankenhausaufenthalt gemeldet wurde, wird nur der erste Krankenhausaufenthalt berücksichtigt. Im Beobachtungszeitraum der vorliegenden Studie wurden 457 PatientInnen mit der Diagnose U07.1 und 1064 mit der Diagnose U07.2 hospitalisiert. Die Analysen folgen dem Vorgehen anderer Studien [16] und ein COVID-19-bedingter Krankenhausaufenthalt liegt vor, wenn einer der beiden Codes gemeldet wurde (1521 Fälle). In zusätzlichen Sensitivitätsanalysen werden die Analysen zudem nur mit im Labor bestätigten Fällen (U07.1) durchgeführt (Tab. 3).

#### *Erwerbssituation*:

Die Krankenkassendaten beinhalten auch Details zur Erwerbssituation ihrer Mitglieder zu Beginn des Beobachtungszeitraums und erlauben die Unterscheidung zwischen „regulärer Erwerbstätigkeit“, „Niedriglohntätigkeit mit Sozialleistungen“, „Arbeitslosigkeit mit Bezug von Arbeitslosengeld 1 (Alg I)“ und „Langzeitarbeitslosigkeit mit Bezug von Arbeitslosengeld 2 (Alg II)“. Eine reguläre Beschäftigung bedeutet, dass der/die Versicherte entweder abhängig beschäftigt oder selbstständig arbeitet. Die zweite Kategorie sind regulär Erwerbstätige mit einem Einkommen, das zu gering ist, um einen Mindestlebensstandard zu gewährleisten, und die daher (neben ihrem Einkommen) zusätzlich Sozialleistungen erhalten (sogenannte ErgänzerInnen). Arbeitslosigkeit mit Bezug von Alg I bedeutet, dass der/die Versicherte arbeitssuchend ist und Leistungen aus der regulären deutschen Arbeitslosenversicherung erhält (etwa 60 % des früheren Nettoeinkommens). Hierunter fallen auch die sogenannten AufstockerInnen, die Alg I erhalten, ihr Einkommen aber aufgrund der geringen Höhe durch Alg II „aufgestockt“ wird. Zur letzten Gruppe, den Langzeitarbeitslosen mit Bezug von Alg II, gehören Arbeitslose, die innerhalb eines bestimmten Zeitraums keine neue Arbeit finden und ein einheitliches eingeschränkteres Langzeitarbeitslosengeld erhalten, die Grundsicherung für Arbeitssuchende (Alg II). Der Zeitpunkt, ab dem Alg II erhalten wird, hängt hierbei vom Alter und der Dauer vorheriger Beitragszeiten zur Arbeitslosenversicherung ab. Der Bezug von Arbeitslosengeld geht in allen Fällen mit einem niedrigeren Einkommen einher, insbesondere im Falle von Alg II, und stellt ein erhebliches Armutsrisiko in Deutschland dar.

### Datenschutz und Ethik

Die hier verwendeten Daten sind Teil der routinemäßigen Datenerhebung von Krankenhäusern und Versicherungsträgern. Durch die Aggregation sind die Daten vollständig pseudonymisiert und ermöglichen keine Rückschlüsse auf die Versicherten. Die Auswertungen erfolgten nach Besprechung aller AutorInnen ausschließlich durch die AOK Rheinland/Hamburg, in enger Abstimmung und mit Genehmigung der Datenschutzbeauftragten der AOK Rheinland/Hamburg.

### Statistische Methoden

Neben deskriptiven Verfahren zur Beschreibung der Studienpopulation kommen multiple logistische Regressionsmodelle für binäre abhängige Variablen zum Einsatz, mit COVID-19-Krankenhausaufenthalt im Beobachtungszeitraum als abhängiges Merkmal. Die Ergebnisse werden als Odds Ratios und Konfidenzintervalle (95 %) präsentiert, die aufgrund der niedrigen Inzidenz als geeignete Schätzer für relative Risiken gelten können. Neben unadjustierten Modellen werden für Geschlecht und Alter (linear und quadratisch) adjustierte Modelle geschätzt. Zudem werden Modelle getrennt für Männer und Frauen berechnet, einschließlich eines formalen Interaktionstests auf Grundlage des gesamten Samples (Vergleich der Modellgüte zwischen einem Modell mit und ohne Interaktionsterme). Alle Berechnungen wurden seitens der AOK Rheinland/Hamburg mit SAS 7.15 durchgeführt, die Abbildung wurde mit Stata 16.1 erzeugt.

## Ergebnisse

Tab. 1 zeigt, dass die Studienpopulation von 1.288.745 Versicherten etwas mehr Männer als Frauen umfasst. Der Altersdurchschnitt beträgt 42 Jahre (nicht in der Tabelle gezeigt). Über 2 Drittel der Versicherten ist regulär erwerbstätig und knapp ein Viertel ist langzeitarbeitslos. Arbeitslosigkeit mit Bezug von Alg I und Niedriglohntätigkeit mit Sozialleistungen sind weniger verbreitet. Insgesamt hatten im Beobachtungszeitraum (01.01. bis 18.06.2020) 1521 Versicherte einen Krankenhausaufenthalt, bei dem COVID-19 dokumentiert wurde. Dies entspricht einer kumulativen Inzidenzrate von 118,02 Fällen pro 100.000 Versicherten, mit höheren Raten bei Männern und Arbeitslosen (insb. Langzeitarbeitslosen).

**Table 1:** 

		COVID-19-Krankenhausaufenthalte	
	Anzahl (Spalten %)	Absolut	Pro 100.000 Versicherten^a^
*Geschlecht*			
Weiblich	570.034 (44,2)	581	101,92
Männlich	718.711 (55,8)	940	130,79
*Erwerbssituation*			
Reguläre Erwerbstätigkeit	923.089 (71,6)	901	97,61
Niedriglohntätigkeit mit Sozialleistungen	35.531 (2,8)	42	118,21
Arbeitslosigkeit mit Alg I	16.560 (1,3)	24	144,93
Langzeitarbeitslosigkeit mit Alg II	313.565 (24,3)	554	176,68
*Gesamt*	1.288.745 (100,0)	1521	118,02

^a^nicht altersstandardisiert

**Figure 1:**
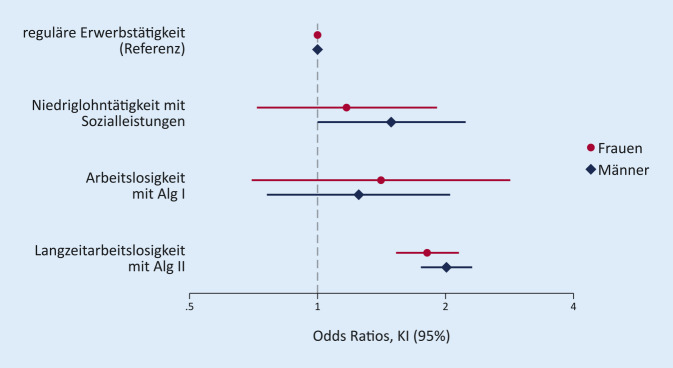

Diese Unterschiede zeigen sich auch in den Ergebnissen der Regressionsanalysen in Tab. 2. So ist die Wahrscheinlichkeit eines Krankenhausaufenthalts nach Kontrolle von Geschlecht und Alter (Modell 2) für Langzeitarbeitslose um 1,94-mal größer als für regulär Erwerbstätige (Referenzgruppe). Auch für Arbeitslosigkeit mit Bezug von Alg I (1,29-mal höher) und für Niedriglohntätigkeit mit Sozialleistungen (1,33-mal höher) sind die Wahrscheinlichkeiten jeweils erhöht. Diese Befunde bestätigen sich auch in zusätzlichen Sensitivitätsanalysen, die sich auf im Labor bestätigte COVID-19-Fälle (ICD-Code U07.1) beschränken (Tab. 3). Abb. 1 zeigt die Zusammenhänge getrennt für Männer und Frauen und zeigt, dass die Ergebnisse für beide Geschlechter ähnlich sind, insbesondere im Falle der Langzeitarbeitslosigkeit. Zwar finden sich leichte Unterschiede im Falle der Niedriglohntätigkeit (etwas stärker für Männer), doch sind die Unterschiede insgesamt statistisch nicht signifikant (Chi^2^ (3) = 0,92, *p* = 0,82).

**Table 2:** 

	Modell 1 unadjustiert OR (KI 95 %)	Modell 2 adjustiert für Geschlecht und Alter OR (KI 95 %)
Reguläre Erwerbstätigkeit (Referenz)	1	1
Niedriglohntätigkeit mit Sozialleistungen	1,21 (0,89–1,65)	1,33 (0,98–1,82)
Arbeitslosigkeit mit Alg I	1,49 (0,99–2,23)	1,29 (0,86–1,94)
Langzeitarbeitslosigkeit mit Alg II	1,81 (1,63–2,01)	1,94 (1,74–2,15)
Weiblich (Referenz)	–	1
Männlich	–	1,31 (1,18–1,45)
Alter (linear)	–	1,00 (0,97–1,04)
Alter (quadratisch)	–	1,00 (1,00–1,00)

**Table 3:** 

	OR (KI 95 %)
Reguläre Erwerbstätigkeit (Referenz)	1
Niedriglohntätigkeit mit Sozialleistungen	1,81 (1,13–2,92)
Arbeitslosigkeit mit Alg I	1,38 (0,68–2,78)
Langzeitarbeitslosigkeit mit Alg II	1,65 (1,35–2,02)
Weiblich (Referenz)	1
Männlich	1,26 (1,05–1,53)
Alter (linear)	1,07 (1,01–1,14)
Alter (quadratisch)	1,00 (0,99–1,00)

## Diskussion

Versicherte in Arbeitslosigkeit mit Bezug von Alg I oder Versicherte in Langzeitarbeitslosigkeit mit Bezug von Alg II haben im Vergleich zu regulär Erwerbstätigen ein erhöhtes Risiko für einen Krankenhausaufenthalt mit COVID-19 – so lautet das Kernergebnis dieser Studie. Ein erhöhtes Risiko zeigt sich auch für Versicherte in Niedriglohntätigkeiten mit zusätzlichem Bezug von Sonderleistungen. Zudem gelten die Ergebnisse gleichermaßen für Männer und Frauen und auch dann, wenn nur laborbestätigte COVID-19-Fälle betrachtet werden. Damit stehen diese Ergebnisse in Einklang mit früheren Befunden für die H1N1-Pandemie im Jahre 2009/2010 und die saisonale Influenza [5–9, 28, 29]. Zudem bestätigen sie bisherige Studien zu sozioökonomischen Unterschieden bei COVID-19 [10–12], insbesondere zu solchen, die die Schwere eines COVID-19-Krankheitsverlaufs (i.e. Krankenhausaufenthalt oder COVID-19-Mortalität) in Abhängigkeit vom Einkommen [21, 23] oder dem Empfang staatlicher Fürsorge [24] untersucht haben. Allerdings sind diese Studien bisher auf andere Länder beschränkt. Damit erweitert der Beitrag den bisherigen Forschungsstand und liefert erste Hinweise dafür, dass sozioökonomische Unterschiede in Bezug auf schwere Verläufe von COVID-19 mit Krankenhausaufenthalt auch in Deutschland gelten – einem Land mit vergleichsweise freiem Zugang zu medizinischer Versorgung.

Das stärkt die empirische Evidenz für soziale Ungleichheiten bei COVID-19. Unbeantwortet bleibt aber die Frage nach den möglichen Ursachen für diese Unterschiede. In Anlehnung an Quinn und Kollegen, die sich allgemein mit möglichen Verbindungen zwischen sozialen Faktoren und Infektionskrankheiten beschäftigt haben, scheint es hierzu hilfreich, 3 mögliche Erklärungen zu unterscheiden [30, 31].

Eine erste Erklärung könnten *Ungleichheiten in der Exposition* gegenüber dem Virus sein. Beispielsweise bestätigen neuere Studien, dass sozioökonomisch benachteiligte Bevölkerungsgruppen häufiger in Berufen arbeiten, in denen die Wahrscheinlichkeit, mit dem Virus in Kontakt zu kommen, erhöht ist [32, 33]. Ähnlich gilt, dass Personen mit höheren Einkommen vergleichsweise häufiger die Möglichkeit der schützenden Heimarbeit haben (mit Ausnahme von Berufen in der Gesundheitsversorgung). Daneben leben Menschen mit geringerem Einkommen häufiger in ungünstigen Wohnverhältnissen (einschließlich beengter Wohnungen mit höherer Ansteckungsgefahr und möglicher Exposition im öffentlichen Nahverkehr).

Eine zweite Erklärung sind *Ungleichheiten in der Vulnerabilität*. Denn aufgrund existierender gesundheitlicher Ungleichheiten sind sozioökonomisch benachteiligte Bevölkerungsgruppen per se bereits häufiger von Vorerkrankungen und weiteren Risikofaktoren betroffen (bspw. Rauchen oder Übergewicht). Damit sind sie anfälliger für Infektionen bei Exposition und haben im Fall einer Infektion ein höheres Risiko für schwere Erkrankungsverläufe. Neuere Studien betonen an dieser Stelle auch die Rolle der umweltbezogenen Schadstoffexposition (z. B. erhöhte Luftverschmutzung in ärmeren Vierteln) mit ihren Auswirkungen auf gesundheitliche Vorbelastungen [34, 35].

Eine dritte Erklärung sind *Ungleichheiten in der Versorgung*. Das bezieht sich nicht nur auf die grundsätzliche Frage nach dem möglichen Zugang zu medizinischer Versorgung und der Erreichbarkeit medizinischer Einrichtungen [36]. Damit sind auch seltenere Testmöglichkeiten für ärmere Bevölkerungsgruppen [37] und eine möglicherweise verspätete Inanspruchnahme im Falle einer Erkrankung gemeint [38].

Eine klare Antwort auf die Frage, warum sozioökomische Ungleichheiten bei COVID-19-Krankenhausaufenthalten im hier untersuchten Kollektiv existieren, bleibt aber schwierig und komplex. Die oben genannten Gründe liefern sicherlich wichtige Anhaltspunkte. Doch wirken sie gewiss nicht einzeln, sondern sind miteinander verknüpft. Zudem variiert deren Stellenwert auch entlang der betrachteten sozioökonomischen Merkmale (Einkommen, Bildung und Beruf). So wird die Erforschung der Gründe und ihrer relativen Bedeutung für unterschiedliche sozioökonomische Merkmale – neben einer soliden epidemiologischen Beschreibung von Ungleichheiten – sicherlich Teil zukünftiger Forschung sein müssen.

Für die vorliegende Studie kann davon ausgegangen werden, dass Unterschiede in der Vulnerabilität ein wichtiger Grund sein könnten. Denn wie frühere Studien zeigen, sind sowohl chronische Erkrankungen (bspw. koronare Herzkrankheiten) unter Arbeitslosen häufiger verbreitet als auch verhaltensbezogene Risikofaktoren wie Rauchen oder Adipositas [27, 39, 40] – alles Punkte, die einen schweren Erkrankungsverlauf von COVID-19 bedeuten können [41]. Eine genaue Prüfung dieser Vermutungen war in dieser Studie aufgrund der eingeschränkten Verfügbarkeit von Daten zu Vorerkrankungen allerdings nicht möglich. Eine aktuelle Studie aus England zeigt aber, dass sozioökonomische Unterschiede einer COVID-19-bedingten Mortalität auch dann bestehen bleiben, wenn Vorerkrankungen (inkl. Diabetes, Asthma und koronarer Herzkrankheiten), Rauchen und Übergewicht berücksichtigt werden [16]. Dies deutet darauf hin, dass auch weitere Faktoren für die Vulnerabilität eine Rolle spielen könnten. Ein weiterer möglicher Faktor ist die psychosoziale Belastung durch Arbeitslosigkeit (und innerhalb benachteiligter Berufsgruppen). Es ist gut dokumentiert, dass Arbeitslosigkeit ein starker Stressor ist und dass die physiologische Stressreaktion mit einer Immunsuppression (und einer erhöhten Infektionsanfälligkeit) verbunden ist [42–44].

Unterschiede in der Exposition spielen für die vorliegende Studie vermutlich keine große Rolle. Hierzu liegen bisher auch keine verlässlichen Studien vor, in denen das Infektionsrisiko zwischen Arbeitnehmern und Arbeitslosen verglichen wird. Möglicherweise ist das Infektionsrisiko für Arbeitslose sogar eher geringer, da sie in ihrer Mobilität eingeschränkt sind (z. B. keine Notwendigkeit des Pendelns) und dem Virus nicht durch soziale Kontakte mit Kollegen, Kunden oder Patienten während der Arbeit ausgesetzt sind. Allerdings ist dieser Punkt eher für das Infektionsrisiko selbst und weniger für die Schwere einer Erkrankung einschließlich einer Hospitalisierung wichtig.

Auch die dritte Erklärung (Ungleichheiten in der Versorgung) könnte in unserem Fall weniger bedeutend sein. Das deutsche Gesundheitssystem bietet nämlich einen universellen Zugang zur ambulanten und stationären Versorgung. Trotz dieses freien Zugangs zeigen Studien aus Deutschland jedoch, dass die Inanspruchnahme der Versorgung von ärmeren Bevölkerungsgruppen vergleichsweise selten ist [36, 45]. In unserem Fall könnte dies bedeuten, dass Arbeitslose den Gang in die ärztliche Praxis meiden oder diese verspätet aufsuchen, wodurch es eher zu schweren Erkrankungsverläufen kommt, die einen Krankenhausaufenthalt erforderlich machen.

### Stärken und Schwächen

Diese Arbeit ist die erste Auswertung individueller Daten zu sozioökonomischen Ungleichheiten bei Krankenhausaufenthalten mit COVID-19 für Deutschland auf Basis von Krankenkassendaten und Informationen zur Erwerbssituation. Im Rahmen der Auswertungen konnten alle erwerbstätigen Versicherten der untersuchten Krankenkasse berücksichtigt werden, womit die interne Validität der Ergebnisse hoch ist. Weitere Stärken sind die hohe Fallzahl von über 1 Mio. Versicherten und die Verwendung übermittelter ärztlicher Informationen zu einem COVID-19-Krankenhausaufenthalt. Denn im Vergleich zu einer SARS-CoV-2-Infektion oder COVID-19-Erkrankung (ohne Krankenhausaufenthalt) werden COVID-19-Krankenhausaufenthalte sehr zeitnah und nicht verspätet an den Versicherungsträger übermittelt (bspw. in jedem Quartal durch die Hausarztpraxis). Aus methodischer Sicht erlauben Krankenhausaufenthalte mit COVID-19 somit eine unmittelbare Analyse des Erkrankungsgeschehens. Ein weiterer Vorteil dieser Studie ist die Messung der Erwerbssituation. Sie beruht auf offiziellen standardisierten Aufzeichnungen (und nicht auf selbst berichteten Informationen), die den Bezug von Arbeitslosengeld zuverlässig erfassen und direkte Rückschlüsse auf die Einkommenshöhe der Versicherten ermöglichen. Im Gegensatz zu weiteren sozioökonomischen Merkmalen ist dieser Indikator in den Krankenkassendaten auch einfach zugänglich und erlaubt eine zeitnahe valide Messung. Weitere sozioökonomische Merkmale wie Bildung (häufig lückenhaft) und Einkommen (wird meist je zum Jahres- bzw. zum Beschäftigungsende rückwirkend gemeldet) erscheinen an dieser Stelle weniger zuverlässig. Ein weiterer methodischer Vorteil ist, dass die Erwerbsituation vor der Erkrankung gemessen wurde und COVID-19 eine akute Erkrankung ist. Damit kann eine umgekehrte Kausalität (englisch: „reverse causality“) wohl ausgeschlossen werden, bei der COVID-19-Erkrankte arbeitslos werden.

Gleichzeitig hat diese Arbeit wichtige Einschränkungen. So stammen die Daten von einem gesetzlichen Versicherungsträger (AOK Rheinland/Hamburg) mit Daten, die auf bestimmte Regionen in Deutschland beschränkt sind. Auf Basis dieser Studie können also keine allgemeinen Aussagen für ganz Deutschland getroffen werden. Zudem ist der Anteil an Arbeitslosen unter den Versicherten der AOK Rheinland/Hamburg naturgemäß höher, da Personen, die arbeitslos werden und Alg I oder Alg II erhalten, häufig vom Leistungsträger bei der AOK angemeldet werden. Dies erklärt auch den hohen Anteil an Langzeitarbeitslosen in dieser Studie (fast 25 % der Studienpopulation). Denkbar ist in diesem Zusammenhang auch, dass die gezeigten sozioökonomischen Unterschiede – zwar in der Tendenz gleich – aber in der allgemeinen Erwerbsbevölkerung anders ausfallen als bei den hier betrachteten Versicherten. Vielleicht sind sie sogar ausgeprägter, da Privatversicherte (eine sozioökonomisch weniger benachteiligte Gruppe) in der vorliegenden Studie gar nicht vorkommen. Eine weitere Einschränkung ist, dass Krankenkassendaten (wie die meisten administrativen Daten) häufig nur begrenzte Informationen zu soziodemografischen und sozioökonomischen Faktoren enthalten und dass es wünschenswert gewesen wäre, weitere Faktoren aufzunehmen. Studien aus den USA und Großbritannien zeigen etwa [46–48], dass bestimmte ethnische Minderheiten (z. B. AfroamerikanerInnen in den USA [47]) einen vergleichsweise schwereren Erkrankungsverlauf haben und dass Ethnizität auch stark mit sozioökonomischen Merkmalen verstrickt ist (sogenannte Intersektionalität). Doch sind Informationen zu Migrationshintergrund oder zu Ethnizität kein Teil der Krankenkassendaten. Ähnlich wäre es sicherlich wünschenswert, neben COVID-19-Krankenhausaufenthalten auch ambulant behandelte COVID-19-Erkrankungen betrachten zu können. Denn die meisten Personen mit einer COVID-19-Diagnose werden außerhalb des Krankenhauses behandelt. Eine große Zahl von COVID-19-Diagnosen (ohne Krankenhausaufenthalt) bleibt in unserer Studie daher wahrscheinlich nicht berücksichtigt. Die vorliegende Studie ermöglicht also keine Aussagen über die Gesamtinfektionsrate. Vielmehr liegt der Fokus auf der Schwere einer Erkrankung bzw. auf COVID-19-Erkrankungen, die einen Krankenhausaufenthalt erforderlich machen. Schließlich sollte in weiterführenden Studien auch die genaue Dauer der Arbeitslosigkeit berücksichtigt werden. Je nach Alter und erfolgter Beitragszahlungen kann der Bezug von Alg I beispielsweise zwischen 6 und 24 Monaten variieren. Auch bleibt in den Daten unklar, wie lange eine Person bereits Alg II erhält. Die Dauer einer Arbeitslosigkeit kann daher innerhalb der betrachteten Gruppen variieren.

### Schlussfolgerung

Zusammenfassend liefert diese Studie erste empirische Hinweise auf sozioökonomische Unterschiede bei Krankenhausaufenthalten mit COVID-19 für Deutschland. Spezifisch zeigt sich, dass Versicherte in Kurz- oder Langzeitarbeitslosigkeit im Vergleich zu regulär Erwerbstätigen ein erhöhtes Risiko für einen COVID-19-Krankenhausaufenthalt haben. Wenn dieser Befund sich auch in zukünftigen Studien zeigt – mit alternativen Merkmalen zur Bestimmung der sozioökonomischen Position (z. B. berufliche Position oder Einkommen) sowie anderen COVID-19-Outcomes (bspw. Infektionsrisiko, intensivmedizinische Versorgung, Beatmung oder Mortalität) –, dann unterstreicht dies die Bedeutung sozioökonomischer Merkmale auch bei Infektionskrankheiten. Diese sollten – ebenso wie Alter und Vorerkrankungen – zur Bestimmung von Hochrisikogruppen und zur Entwicklung von Infektionsschutzmaßnahmen beachtet werden [11, 49, 50]. Darüber hinaus unterstreicht unsere Studie, wie wichtig es ist, die Datenlage in Deutschland zu verbessern und sozioökonomische Merkmale zu berücksichtigen, um eine Untersuchung sozioökonomischer Unterschiede während der COVID-19-Pandemie zu ermöglichen.
